# Long-term follow-up of patients with vestibular neuritis by caloric testing and directional preponderance calculation

**DOI:** 10.1007/s00405-022-07660-9

**Published:** 2022-09-26

**Authors:** András Molnár, Benjámin Donát Jassoy, Stefani Maihoub, Panayiota Mavrogeni, László Tamás, Ágnes Szirmai

**Affiliations:** 1 Department of Otolaryngology and Head and Neck Surgery, Semmelweis University, Szigony u. 36., 1083 Budapest, Hungary; 2 Faculty of Medicine, Semmelweis University, Üllői út 26., 1085 Budapest, Hungary; 3 Tóth Ilona Health Service Clinical Medical Institute, Görgey Artúr tér 8., 1212 Budapest, Hungary; 4 Department of Voice, Speech and Swallowing Therapy, Faculty of Health Sciences, Semmelweis University, Vas u. 17., 1088 Budapest, Hungary

**Keywords:** Vestibular neuritis, Electronystagmography, Caloric test, Caloric weakness, Directional preponderance

## Abstract

**Objectives:**

This study focuses on the diagnostic precision of caloric testing in detecting vestibular neuritis (VN).

**Materials and methods:**

In this study, 99 patients (36 men, 63 women, mean age: 44.63 years $\pm$ 12.08 SD) with superior VN were involved, and 157 participants with a normal functioning vestibular system were also investigated. All patients underwent a complete neurotological examination, including the caloric test with electronystagmography registration. The canal paresis (CP) and directional preponderance (DP) values were analysed.

**Results:**

A VN on the right side was diagnosed in 31.3% and on the left side in 68.7%. When the CP parameters between the control and VN patients were contrasted, a statistically significant difference was observed (*p* < 0.00001*, Mann–Whitney *U* test), indicating higher values in the latter group. The prediction of VN based on the CP value was successful in 71%, and statistical analysis indicated a significant result [*p* < 0.0001*; OR: 5.730 (95% CI 3.301–9.948)]. The DP values were also significantly higher in the VN group (*p* < 0.00001*). The prediction of VN according to the DP value was successful in 69.8%. A significant result was also observed in this case [*p* < 0.001*; OR: 4.162 (95% CI 2.653–8.017)]. When both CP and DP were considered, a predictive value of 84.8% with a significant outcome [*p* < 0.0001*; OR: 82.7 (95% CI 28.4–241.03)] was detected.

**Conclusions:**

Including the CP and DP parameters of the caloric test, VN could be detected in around 85%. Therefore, the caloric helps diagnose the disorder, but both parameters must be considered.

## Introduction

The background of vestibular neuritis (VN) is unilateral deafferentation of the peripheral vestibular system, resulting in sustained acute rotatory vertigo for more than 24 hours (i.e., acute vestibular syndrome), accompanied by vegetative symptoms, without any cochlear symptoms (i.e., hearing loss or tinnitus) or focal neurological deficits. During examinations, spontaneous horizontal or horizontal-torsional nystagmus beating toward the unaffected side and reduced caloric responses on the affected side can be recorded [1]. Ipsilateral caloric weakness is traditionally thought to be the gold standard sign for diagnosing the disorder. Based on previous results, the superior vestibular nerve is the most frequently involved in VN [2]. Inflammation of the vestibular nerve is suspected to be caused by the reactivation of a latent herpes virus infection, most likely caused by herpes virus type 1 (HSV-1) [3]. Nowadays, it is also of great importance that cases have been reported after SARS-CoV-2 infection [4] or COVID-19 vaccination [5]. According to previous research, VN is suspected to be the second most common peripheral vestibular disorder, with an incidence of 3.5–15/100,000 and a nearly equal sex distribution [6]. To objectively assess vestibular functions, electro or videonystagmography is one of the most widely used diagnostic tools in daily neurotological practice [7, 8].

Since VN is a frequent neurotological disorder and there are still questions about its diagnosis and differentiation from central vestibular disorders (i.e., posterior circulation strokes), objective testing is a central problem. It is also an essential issue that due to the relatively limited accessibility of the neurotological diagnostic in many countries, particularly since COVID-19, during which many examinations have been cancelled, these examinations are usually unavailable during acute presentations of disorders. Furthermore, caloric testing may not be used in the acute phase of disorders, as it can be stressful and spontaneous nystagmus can result in inverse caloric nystagmus; therefore, it is possible to misinterpret the results. Consequently, this study focuses on the diagnostic use of electronystagmography (ENG) and caloric testing in diagnosing the disorder, and examining patients by appointment, as there remains a need for an efficient method to diagnose VN objectively.

## Materials and methods

### Participants

A total of 99 patients (36 men, 63 women, mean age: 44.63 years $\pm$ 12.08 SD) who were diagnosed with VN of the superior vestibular nerve were enrolled in the present investigation. Furthermore, 157 participants (50 men, 107 women, mean age: 48.84 years $\pm$ 15.2 SD), who were not complaining about vertigo or dizziness and did not present any vestibular symptoms, were also investigated as a control group. The two groups' basic demographical parameters are presented in Table 1. The inclusion criteria for VN patients included previously documented acute vestibular syndrome, with vertigo lasting at least 24 hours, vegetative symptoms and spontaneous horizontal or horizontal–torsional nystagmus beating toward the healthy side, and aberrations of vestibulospinal reflexes that show on the affected side (i.e., falling or deviation during the Romberg’s and stepping tests), without any cochlear symptoms (i.e., acute hearing loss or tinnitus). The HINTS examination (i.e., head-impulse test, nystagmus and test of skew) was performed on all participants to differentiate between central and peripheral vestibular disorders, which is particularly essential in the acute VN cases. Patients with hearing loss (based on pure-tone audiometry or objective audiometry), tinnitus, middle ear diseases, other peripheral or central vestibular disorders, or neurological symptoms were excluded from the present study. The involvement of the superior vestibular nerve was identified by video-head impulse testing (vHIT) of each semicircular canal on both sides and cervical (c) and ocular (o) vestibular evoked myogenic potentials (VEMP). Only patients suffering from superior VN were included. All patients underwent an audiological examination to exclude any cochlear symptoms, including pure-tone audiometry, tympanometry, and objective audiometry in necessary cases. Vestibular schwannoma and other central vestibular aberrations were excluded by brain MRI. The neurotological examination was not performed in the acute phase of vertigo, but later on an appointment basis, with a mean follow-up time of 13.7 $\pm$ 11.9 months (range: 2—72 months). This study was approved by the Semmelweis University Regional and Institutional Committee for Science and Research Ethics (48/2018).

**Table 1:** Demographical parameters in the VN and control groups

	**VN**	Control group	***p*** value
Age (mean years $\pm$ SD)	44.63 $\pm$ 12.08	48.84 $\pm$ 15.2	0.069*
Gender distribution (men/women)	36/63	50/107	0.55**

*SD* standard deviation, *VN* vestibular neuritis

*Mann–Whitney *U* test

**Chi-square test. Significance level: *p* < 0.05

### Electronystagmography examination

Before the examination, the participants' middle ear status was checked in all cases, using otoscopy and tympanometry. The ENG examination, mainly the caloric test, was not performed if any aberration of the middle ear was detected. Before examination, the ENG system was calibrated in all cases. The patients were lying supine during the examination with their heads tilted 30°, orienting the horizontal canals vertically. An MCU-100 ENG system (ICS Medical Corporation, Schaumburg, Illinois) was applied, and as the first steps of the examination, spontaneous nystagmus and optokinetic eye movements were analysed. In the case of spontaneous nystagmus, its velocities and frequencies were calculated. In addition, a bithermal caloric test was performed, using warm and cold air as stimuli, by applying an air caloric stimulator (NCA-200, CHARTR). Between each irrigation, a 5-min break was taken. After each irrigation, the maximum slow phase velocity of the nystagmus was calculated and the modified Jongkees’ formula was used to determine the parameters of canal paresis (CP) and directional preponderance (DP). CP indicates the percentage loss of unilateral hypofunction, while asymmetry can also result from a hyper reaction of one side, which can be determined using the DP parameter. Normative values were defined based on previous studies, as follows: normal (CP ≤ 20%), intermediate (CP: 21–40%), and severe (CP > 40%) [9], and in the case of DP, a value less than 30% was considered normal [10].

### Statistical analysis

SPSS Statistics V24 software (IBM Corporation, Armonk, NY, USA) was applied for statistical analysis. As the Shapiro–Wilk test indicated a not normal distribution of the data, a non-parametric test (i.e., Mann–Whitney *U* test) was applied to check for significant differences. In addition, logistic regression was also applied. A *p* value below 0.05 was considered statistically significant.

## Results

Table 1 summarises the two investigated groups' basic demographic data (i.e., control and VN groups).

As Table 1 illustrates, the participants were found around 50 years of age, and the gender distribution was almost identical in both groups, indicating a slight female predominance. According to statistical analysis, no significant differences were detected in the cases of age (*p* = 0.069) and sex (*p* = 0.55) of the participants. Therefore, it can be assumed that the two groups are suitable for further analyses. The VN on the right side was diagnosed in 31.3% and on the left in 68.7% of cases. Therefore, a predominance of left side superior vestibular nerve involvement was observed in the current population.

**Fig. 1:**
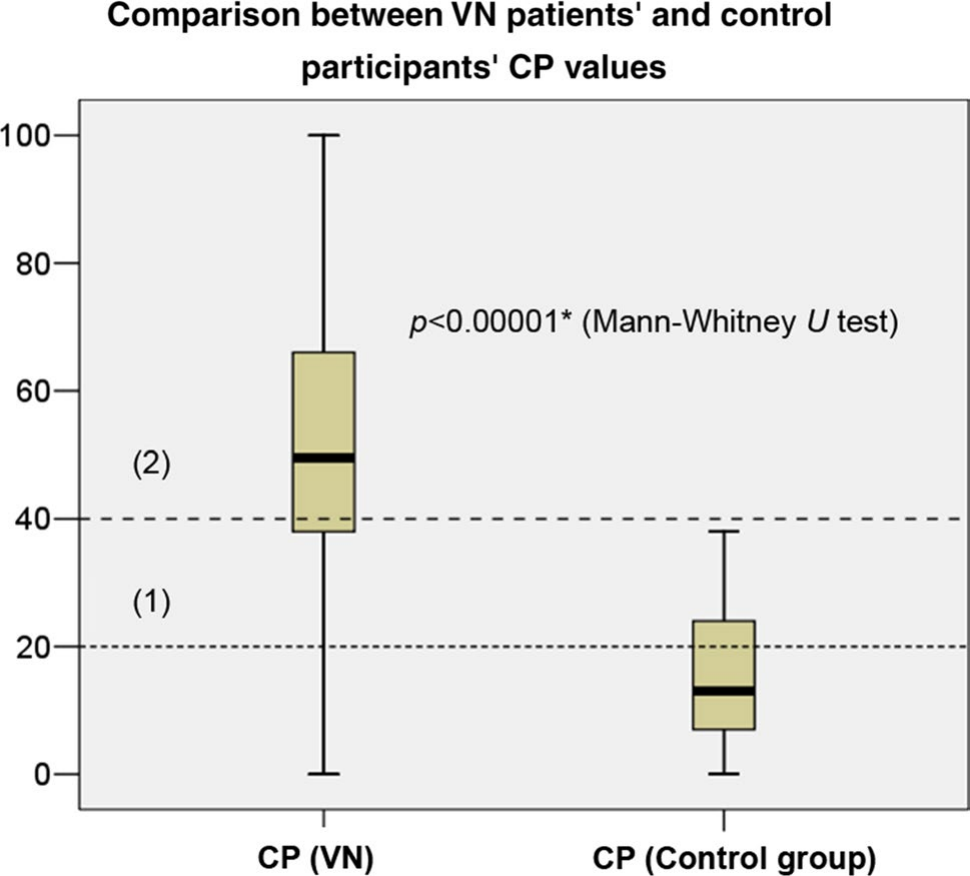
Boxplot illustrating the CP values in the VN and control groups. (1): 20% (CP normal range), (2): 40% (severe CP). Black lines in the boxes: median values, box: the middle 50% of the data, whiskers: upper and lower 25%. The Mann–Whitney *U* test was used to detect differences between the examined parameters (*p < 0.05). The * refers to a statistically significant difference. *CP* canal paresis, *VN* vestibular neuritis

Figure 1 illustrates the CP values detected in the control and VN groups.

The CP data in Fig. 1 indicate an apparent difference between the VN and control participants' CP values, indicating higher values in the VN group. As can be identified from Fig. 1, the difference between the two groups' parameters was statistically significant (*z*-score: -10.73, *p* < 0.00001*). Therefore, it is speculated that significantly higher CP values were observed in the case of VN. Based on the boxes, it appears that most of the CP values in VN cases were found to be in the severe range, while in the control group, they were in the normal range.

As the next analysis step, the prediction values of CP in VN were calculated. The results are displayed in Table 2.

**Table 2:** Prediction of VN based on the CP value

	CP normal	CP pathological
Control group	114 (44.5%)	43 (16.8%)
VN	31 (12.1%)	68 (26.6%)
Overall percentage		71%

*CP≤20%* normal, *CP>20%* pathological, *CP* canal paresis, *VN* vestibular neuritis

As Table 2 reports, a higher ratio of normal CP values was observed in the case of the control group (44.5%) and a higher ratio of pathological values in the VN group (26.6%). This result could also be interpreted as based on the overall percentage value, the prediction of VN was successful in 71%. Even though it could be assumed that this result is less than perfect, using only the CP parameter by itself, the majority of VN and control cases can be identified. In addition, logistic regression was applied to analyse the correlation between the values presented in the table. According to this analysis, a significant correlation was identified [*p* < 0.0001*; OR: 5.730 (95% CI 3.301–9.948)]. This confirms the results given in Table 2, i.e., a significantly higher ratio of normal CP values was detected in the control and a higher pathological ratio in the VN group.

In the next section, an analysis of the DP value is presented. In this case, the results are divided into two parts as well (i.e., DP values in the control and VN groups and DP-based prediction).

**Fig. 2:**
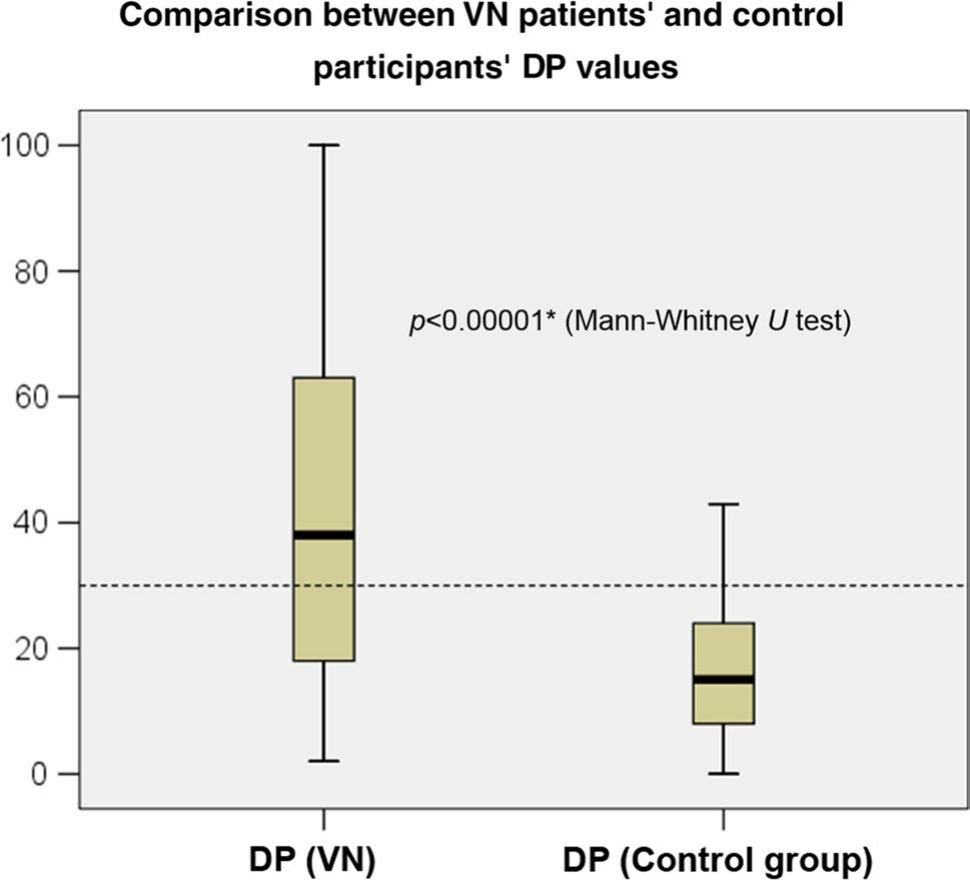
Analysis of the DP values in the VN and control groups. The Mann–Whitney *U* test was used to detect differences between the examined parameters (*p < 0.05). The horizontal interrupted line indicates the normal DP range (i.e., 30%). The * sign refers to a statistically significant difference. *DP* directional preponderance, *VN* vestibular neuritis

From Fig. 2, it can be seen that there is a visible difference between the DP values in the VN and the control groups, suggesting higher DP values in the case of patients with VN. When statistical analysis using the Mann–Whitney *U* test was performed, a significant difference (*z*-score: − 6.57, *p* < 0.00001*) between the two groups was also observed in this case. In addition, an inspection of Fig. 2 represents that most values in the VN group were classified as pathological, while the control group's parameters were found to be in the normal range. From these results, it seems plausible that a relative hyperfunction of the contralateral vestibular system based on the DP value of the caloric test may also be a sign of detecting VN.

DP prediction values were also calculated and the results are listed in Table 3.

**Table 3:** VN prediction values based on the DP parameter

	DP normal	DP pathological
Control group	124 (48.4%)	33 (12.8%)
VN	44 (17.2%)	55 (21.6%)
Overall percentage		69.8%

*DP* directional preponderance, *VN* vestibular neuritis

From Table 3, it can be observed that a pathological DP value occurred more frequently in the VN than in the control group. The comparison of the CP results as reported in Table 2. shows that the distribution was a bit lower, but the prediction of VN based on the DP parameter was successful in around 70% of the cases. When statistical analysis was performed using logistic regression, a significant result was also obtained in this case [*p* < 0.001*; OR: 4.162 (95% CI 2.653–8.017)].

To analyse the prediction value of the two parameters in combination, the analysis detailed in Table 4 was carried out.

**Table 4:** Prediction value of the CP and DP parameters

	CP and DP normal	CP/DP pathological
Control group	122 (47.7%)	35 (13.6%)
VN	4 (1.6%)	95 (37.1%)
Overall percentage		84.8%

In this case, the ipsilateral CP and contralateral DP values were considered. When one of the CP/DP parameters was found to be in the pathological range, the case was classified as pathological, as demonstrated in the table

*CP* canal paresis, *DP* directional preponderance, *VN* vestibular neuritis

As Table 4 reveals, when the CP and DP parameters were also considered, an overall NV prediction of around 85% was calculated. According to logistic regression, a significant outcome was observed [*p* < 0.0001*; OR: 82.7 (95% CI 28.4–241.03)]. Comparing Tables 2, 3, and 4 shows that the highest prediction value was identified in this case. This result supports the idea that the parameters appear to provide complementary information; therefore, both parameters should be considered.

## Discussion

The present investigation focused on the diagnostic use of the caloric test with ENG registration in diagnosing VN. This study included many VN cases with a predominance on the left side. This distribution is interesting, as it is inconsistent with previous results that did not demonstrate an asymmetric distribution in VN [11]. However, other peripheral vestibular disorders, such as Ménière’s disease, with tinnitus and sudden deafness, occur more frequently in the inner ears of the left side, according to previous research results [12, 13]. Since VN is a frequent disorder and its differentiation from central vestibular disorders is an important issue; moreover, the exact diagnostic criteria and a diagnostic guideline are still missing, finding an objective test to help the diagnostic method is essential. Based on our analyses, around 85% of the VN cases can be detected, using the caloric test, including the CP and DP parameters. This ratio is not ideal but can be stated as a fair result. This result is significant, as the caloric test is still a widely used method in daily neurotological practice. Other parameters of the ENG examination (e.g., spontaneous nystagmus or parameters of optokinetic eye movements) are also of great importance in everyday practice, but these were not part of the present investigation.

One of the limitations of the caloric test is that the caloric test can only evaluate the functioning of the horizontal semicircular canals and, hence, the superior parts of the vestibular nerve. However, superior VN is the most common type of disorder, with significantly higher rates (e.g., 97.7%) of horizontal canal involvement [14]. Consequently, most VN cases can be identified using the caloric test. However, this fact should be mentioned as a limitation of the caloric test; therefore, the caloric test should be used as a part of a complete neurotological examination, including other objective methods (e.g., vHIT, VEMP, etc.). Although medical imaging methods are widely used in everyday medical practice, their results in VN are still questionable. A study on vestibular nerve atrophy magnetic resonance imaging (MRI) in VN observed a statistically significant decrease in the cross-sectional area and height of the vestibular nerve, compared to the parameters of the contralateral vestibular nerves [15]. However, another investigation showed that only half of the participants had vestibular nerve atrophy and the others did not [16]. Therefore, it can be concluded that vestibulo-ocular reflex (VOR) examinations are still of great importance, e.g., using the caloric test. In addition, these methods can also objectively calculate the severity of function loss, which can be significant given patient follow-up.

A previous study contrasted the caloric test results and c-VEMP in VN. Their results showed a reduced caloric response in all cases, although 88% showed bilateral normal responses during the c-VEMP examination. These results also confirm that VN affects mainly the superior vestibular nerve; therefore, the caloric test can be more informative than, for example, the c-VEMP examination, which is especially advantageous in the case of inferior VN [17]. However, o-VEMP was not used in that study. Furthermore, the two methods (i.e., caloric testing and VEMP) examine different parts of the vestibular system. As mentioned previously, superior VN is more common than involvement of the inferior branch (48.1% vs 12%) [18]. The distribution, as previously observed, can be explained by several theories, i.e., the direct viral inflammation selectively affecting the superior branches or the longer and narrower bony channel of the superior parts; therefore, making the superior parts more vulnerable to oedema [19]. On the other hand, a recent study concluded that even though the narrower bony channel contributes to the more common involvement of the superior nerve, other factors, i.e., innervation redundancy of the posterior semicircular canals and the saccule and anastomoses between the facial and superior vestibular nerve, which is important in the case of reactivation of herpes viruses, are also essential for the involvement of the superior branch [20]. The clinical importance of this fact is the more common occurrence of superior VN, in which case the one-sided aberration can be registered using the caloric test.

It is also of great importance that spontaneous recovery can occur after an acute vestibular syndrome. Consequently, performing vestibular tests too early or late may not provide representative parameters regarding vestibular functioning. In the present investigation, patients were not examined in the acute phase of vertigo, but rather on an appointment basis; notwithstanding, a high rate of abnormal results was obtained. A previous investigation reported reduced caloric responses in VN with long-term follow-up. Finally, it was reported that after 1 month, 90%, after 6 months, 80%, while after 5 or 10 years, 50% of the participants presented a reduced caloric response in that study. This result indicated that the hypofunction of the horizontal semicircular canals is not always repaired, despite a possible improvement in subjective symptoms [21]. In the present investigation, a permanently registered reduced caloric response was also observed, as 69% of the caloric responses were pathological in the VN group, with a mean follow-up time of 13.7 months of symptoms. Furthermore, previous research reported that persistent canal paresis resulted in significant atrophy of the superior vestibular nerve observed by MRI. However, since half of the patients did not show significant differences compared to the control group, these results should be confirmed, including a larger sample size [16]. Another study stated that the CP results were pathological in 76% of the follow-up stage, indicating a persistent CP value in the VN. In addition, using the CP parameter, it was possible to detect the lesion side of VN in all cases, regardless of other examinations with normal outcomes in that study. Furthermore, when CP was in the normal range, the other applied examinations (i.e., vibration-induced nystagmus, head-shaking nystagmus and subjective visual vertical tests) could predict VN [22]. The present study reported a nearly equal distribution of persistently reduced caloric responses.

According to the results of the current investigation, when an asymmetrical function of the vestibular system occurs, a hypofunction of the ipsilateral side and a hyperfunction of the contralateral side may occur simultaneously. Therefore, the DP parameter of the caloric test is also helpful. On the other hand, there is relatively limited data in the literature on vestibular hyperfunction in VN. A study revealed that during the acute presentation of VN, no reductions in caloric responses were observed; only a directional preponderance was detected. According to the follow-up times in the current investigation, a persistent hyperfunction of the horizontal semicircular canals in VN may be present. Furthermore, no corrective saccades were observed during the head-impulse test, while spontaneous nystagmus was presented and MRI findings were normal in that study. Therefore, the authors stated that a negative head-impulse test with spontaneous vestibular symptoms might not be symptoms of a central vestibular disorder [23]. This finding suggests that there are remaining questions regarding the differentiation of central and peripheral vestibular disorders. In the future, care should also be taken concerning differential diagnosis. Another investigation contrasted the caloric test CP and the manual rotational VOR test DP values. According to their results, the two parameters showed a significant correlation. Furthermore, in the case of the DP value, a wide variation was detected when the CP value was found to be between 40% and 80%. Hence, it was concluded that the DP value of manual rotational testing is helpful when the CP is in the 40–80% range. However, manual rotational testing is nowadays limitedly used in daily neurotological practice [24].

Nowadays, the vHIT is widely used as it is a relatively easy method and can also be used at the bedside. However, the literature shows a limited correlation between vHIT and the results of the caloric test, which is based on the fact that the two methods apply two different stimuli (i.e., low- and high-frequency stimuli) with different targets in the vestibular system (i.e., regular or irregular fibres and Type I or II hair cells). For example, in a recent study, a dissociation was observed between the results of the caloric and horizontal vHIT tests. In conclusion, an abnormal caloric test and a normal vHIT can indicate a central disorder, while a reversed distribution can indicate a peripheral vestibular disorder. Consequently, vHIT cannot replace the caloric test in daily practice [25]. Another study did not observe a significant correlation between the caloric test, vHIT and the results of the Dizziness Handicap Inventory Questionnaire [26]. Another study also did not observe a correlation between caloric testing and vHIT results in VN and Ménière’s disease. However, 96.3% of the VN cases showed abnormal caloric test results in that study, referring to a significantly higher percentage of the CP value than in the control group. However, that study did not consider the DP value [27].

## Conclusions

Including the CP and DP parameters of the caloric test, VN could be detected in around 85%. Therefore, the caloric helps diagnose the disorder and follow-up patients, but both parameters must be considered. Future studies will be carried out on the other ENG parameters and their use in diagnosing VN.
